# Gender Differences in Psychological Outcomes Following Surf versus Hike Therapy among U.S. Service Members

**DOI:** 10.3390/ijerph21020241

**Published:** 2024-02-19

**Authors:** Lisa H. Glassman, Nicholas P. Otis, Kim T. Kobayashi Elliott, Betty Michalewicz-Kragh, Kristen H. Walter

**Affiliations:** 1 Health and Behavioral Sciences, Naval Health Research Center, San Diego, CA 92106, USA; nicholas.p.otis.ctr@health.mil (N.P.O.); kristen.h.walter.civ@health.mil (K.H.W.); 2 Leidos, Inc., San Diego, CA 92121, USA; 3 Department of Public Health, Naval Medical Center San Diego, San Diego, CA 92134, USA

**Keywords:** exercise, physical activity, depression, outdoor recreation programs, nature exposure, outdoor activity, military

## Abstract

Background: Surf and hike therapies have demonstrated effectiveness as adjunct interventions for service members with major depressive disorder (MDD). This study explores gender differences in intervention outcomes following a pragmatic, randomized controlled trial of Surf and Hike Therapy for service members with MDD (*N* = 96; men, *n* = 46; women, *n* = 50). Methods: Clinician-administered and self-report measures (depression, anxiety, positive affect, negative affect, resilience, and pain) were completed at preprogram, postprogram, and 3-month follow-up; brief measures (depression/anxiety and positive affect) were completed before and after each session. Results: Multilevel modeling results showed that anxiety decreased from pre- to postprogram and significantly differed by gender (B = −2.26, *p* = 0.029), with women reporting greater reductions. The remaining outcomes from pre- to postprogram demonstrated significant improvements that did not differ by gender (*p*s = 0.218–0.733). There were no gender differences through follow-up (*p*s = 0.119–0.780). However, within sessions, women reported greater improvements in depression/anxiety (B = −0.93, *p* = 0.005) and positive affect (B = 3.73, *p* = 0.001). The change in positive affect scores within sessions was greater for women in Hike Therapy compared to men (*p* = 0.016). Conclusions: Overall, results demonstrate that both genders benefit from adjunctive Surf and Hike Therapies, but women exhibit a better response in terms of longer-term anxiety and immediate psychological outcomes.

## 1. Introduction

Major depressive disorder (MDD) is one of the most prevalent psychological conditions in the United States [1,2]. U.S. active duty service members experience MDD at relatively high rates that have increased over time, including a 67% increase in positive depression screenings following the start of the COVID-19 pandemic [3,4,5,6]. The impact of MDD on military personnel and the military is far-reaching. Among veteran and activity duty populations, MDD is correlated with physical and psychological issues, including suicidality, substance use, and medical conditions (e.g., coronary heart disease; [7,8,9,10]), and MDD is associated with increased service-related disability and hospitalizations [11,12]. Given the significant and comprehensive impact that MDD has upon service member health and functioning, developing and delivering effective treatments is a priority for the Department of Defense. Although a variety of evidence-based therapies exist (e.g., cognitive behavioral therapy, behavioral activation, and antidepressant medications), it is estimated that 50–66% of individuals do not respond adequately to these interventions [13,14,15].

Exercise has demonstrated effectiveness in treating the symptoms of MDD. Physical activity has been associated with improved mood, sleep, and quality of life among individuals with depression [16,17]. A recent meta-analysis combined findings from 25 randomized controlled trials (RCTs) that compared exercise therapy to control conditions for individuals with depression and found that exercise therapy significantly improved symptoms [18]. Surf and hike therapies, in particular, have been shown to be beneficial among active duty populations, where exercise therapies may be especially useful given their emphasis on physical health while avoiding the stigma associated with seeking traditional mental health interventions [19,20]. Among service members with MDD, surf and hike therapies have demonstrated significant, immediate improvements in pain and positive affect following sessions, as well as longer-term improvements in depression, anxiety, positive affect, negative affect, psychological resilience, and social functioning up to three months following intervention completion [21,22]. In general, surf and hike therapies have comparable benefits on psychological outcomes; they are both physical activities that include socialization and occur in the natural environment, which has been shown to have a positive influence on depression symptoms [23,24]. However, some differences (e.g., within-session positive affect improvements) have emerged from direct comparisons of surf and hike therapies and favor surf therapy [21,22]. Researchers posit that surfing may result in better improvements in positive affect outcomes because it combines outdoor activity and water-based environments (i.e., “blue space”), which may result in enhanced psychosocial functioning, self-esteem, and mood [25,26,27]. Taken together, current research supports the utility of surf and hike therapies for improving psychological health and functioning for service members with MDD.

Although the benefits of surf and hike therapies have been established among individuals with MDD, questions remain regarding whether there are differential responses to these interventions based on individual characteristics. Gender differences represent an area in need of further attention for a couple of reasons. First, research suggests that there are gender differences in the expression of MDD symptoms. For example, compared to men, women with MDD are more likely to report sexual impairments and physical pain and experience longer depressive episodes, with an increased likelihood of a chronic course [28,29]. Further, there is emerging evidence that there are gender differences in the impact of exercise across mental health symptoms [30,31,32]. One review, which synthesized findings from meta-analyses that examined the impact of exercise and physical activity on depression, reported that men with depression or anxiety may benefit more than women following exercise interventions [32]. Taken together, these findings highlight the importance of additional research on gender differences in the impact of exercise interventions among individuals with depression.

Additionally, examining gender differences for exercise intervention outcomes is especially important within the U.S. active duty population, where male culture tends to dominate [33,34,35]. Despite representing 17% of the military population [36], women often report feeling marginalized [33,37] and experience high levels of perceived stress related to their gender [38]. However, there have been few examinations of gender differences following interventions in this population. To our knowledge, only one study has examined gender differences following an exercise intervention among service members. This study showed that, on average, women reported greater improvements in immediate depression/anxiety and positive affect scores compared to men following surf therapy [30]. Although a single study, these results contradict the nascent body of literature in non-military samples, which has found that men benefit more than women following exercise therapies [32]. Further research is needed in the active duty population to determine whether gender moderates exercise outcomes, as these findings can inform clinical care and service delivery.

The current study extends the literature by examining gender differences in psychological outcomes following two exercise interventions, surf and hike therapies, among active duty service members with MDD. More specifically, analyses will examine whether there are gender differences in depression, anxiety, positive affect, negative affect, and pain from preprogram through the three-month follow-up period, as well as differences in depression/anxiety and positive affect before and after each exercise session. Furthermore, this study will explore whether gender differences are contingent on exercise intervention type (i.e., surf versus hike therapy). Given that one study among service members found that women experienced greater improvements in depression/anxiety following surf therapy, we hypothesize that women will experience greater benefits compared to men in our sample. This study is a secondary data analysis of a randomized controlled trial published elsewhere [22].

## 2. Materials and Methods

### 2.1. Intervention Programs

The Surf and Hike Therapy Programs were offered at Naval Medical Center San Diego (NMCSD) as an optional component of standard care for psychological and physical rehabilitation. Sessions ran once weekly for 3–4 h per session over 6 weeks and were conducted in a cohort format; 20 service members were able to participate per cycle. All sessions were scheduled as medical appointments. Surf Therapy took place at a single public beach in San Diego, and Hike Therapy took place at multiple locations throughout San Diego County. Both programs were led by Master’s-level program managers specializing in exercise physiology and recreation therapy. The Surf and Hike Therapy programs did not include a psychotherapy component; rather, participation in the exercise was considered the therapeutic element. For further details, see the parent study [22].

### 2.2. Participants

Ninety-six active duty service members diagnosed with MDD were recruited as part of an RCT [22]. Service members were assessed for psychiatric disorders using the Mini International Neuropsychiatric Interview version 7.0 (MINI-7) [39]. Exclusion criteria encompassed previous participation in the NMCSD Surf or Hike Therapy programs and a lack of medical clearance (e.g., seizure disorder) from an NMCSD provider to participate in such programs. There were no restrictions on other treatments (e.g., psychotherapy and pharmacotherapy) while participating in the programs; however, data on the use of other treatments were collected and analyzed. For the current analysis, participants were categorized into two groups a posteriori based on their self-identified gender at the preprogram assessment (women, *n* = 50; men, *n* = 46). See Table 1 for additional preprogram sample characteristics.

### 2.3. Study Design and Procedure

After referral to the parent study, service members were assessed for eligibility by an assessor who was blinded to the exercise intervention assignment. If service members met diagnostic MDD criteria based on the MINI-7, blocked randomization was used to randomly assign each participant to Surf or Hike Therapy. Participants in the Surf Therapy program were eligible to receive an optional yoga session prior to the start of each surf session. Following completion of this preprogram assessment, service members then participated in either Surf or Hike Therapy for 6 weeks and were re-assessed within 2 weeks of program completion, as well as at 3-month follow-up. To capture the immediate effects of participation on psychological outcomes, participants also completed a brief assessment before and after each exercise therapy session. All study procedures were approved by the NMCSD Institutional Review Board and performed in compliance with all applicable federal regulations governing the protection of human subjects.

### 2.4. Measures

Depression symptom severity was assessed with both clinician-rated and self-reported measures. The Montgomery–Åsberg Depression Rating Scale (MADRS) [40] was used as a semi-structured, clinician-rated measure. The MADRS consists of 10 items on a 0–6 scale (range: 0–60), with higher scores indicating greater depression severity. Clinically meaningful change on the MADRS is reflected by a 6-point change on the measure [41]. Assessor intraclass correlation was excellent (0.91). Self-reported depression symptom severity was assessed using the 9-item Patient Health Questionnaire (PHQ-9) [42]. The PHQ-9 measure assesses depression symptoms over the last two weeks on a 4-point scale (range: 0–27). Higher scores indicate greater depression-symptom severity. A 5-point change on the PHQ-9 reflects a clinically meaningful change in depression [43].

Anxiety symptom severity was measured using the 7-item Generalized Anxiety Disorder Scale (GAD-7) [44]. Symptoms over the last two weeks were reported on a 0–3 scale, resulting in a total score of 0–21, where higher scores reflect greater anxiety severity. A 4-point change on the GAD-7 indicates clinically meaningful changes in anxiety [45]. The Positive and Negative Affect Schedule (PANAS) measures participant affect [46]. The PANAS can be separated into two subscales for positive affect (PAS) and negative (NAS) affect. These subscales contain 10 emotions each, which are rated from 0–4 and summed for a total score ranging from 0–40. Higher scores suggest higher levels of positive affect and negative affect, respectively. Resilience was measured using the 4-item Response to Stressful Events Scale (RSES-4) [47]. This instrument is scored from 0–4, and higher sum scores indicate greater resilience in response to stressful events. Pain was assessed using the single-item Numerical Pain Rating Scale (NPRS) [48], which ranges from 0–10, with a 10 signifying the greatest pain. All of the aforementioned scales were measured at preprogram, postprogram, and 3-month follow-up.

Immediately before and after exercise sessions, depression/anxiety was measured using the 4-item Patient Health Questionnaire (PHQ-4) [49]. This brief scale consists of two depression items from the PHQ-9 and two anxiety items from the GAD-7. Items are scored from 0–3 and summed, resulting in a severity score from 0–12 where higher scores denote greater symptom severity. Positive affect was also measured before and after exercise sessions with the PAS.

During the preprogram assessment, participants provided data on demographics, service characteristics, concurrent treatment utilization, and physical activity. The 7-item International Physical Activity Questionnaire-Short Form (IPAQ-SF) was used to assess preprogram physical activity [50]. The IPAQ-SF score is summed according to IPAQ manual instructions [51] and indicates the frequency and intensity of physical activity over the last week in metabolic equivalent minutes (MET mins) via three categories that align with World Health Organization guidelines: low (<600 MET mins/week), medium (600–2999 MET mins/week), and high (≥3000 MET mins/week).

### 2.5. Statistical Analysis

Analyses were conducted with IBM SPSS Statistics (Version 29). The data were analyzed as intent-to-treat. Descriptive statistics established sample and preprogram characteristics; chi-square tests of association and independent samples *t*-tests examined differences in sample characteristics by gender. Multilevel models (MLMs) were used to analyze outcome differences by gender over time.

Both longitudinal (i.e., preprogram, postprogram, and 3-month follow-up) and within-session (i.e., presession and postsession) MLMs used a step-up model-building process; logical covariance matrices were compared and selected based on model fit according to the Akaike Information Criterion with respect to the number of parameters specified. In the initial models, independent variables that were neither statistically significant nor theoretically relevant to the purpose of the gender analyses were removed. All final models used restricted maximum likelihood to account for missing data.

For longitudinal analyses, the intercept was set as a random effect of subject with a diagonal covariance matrix. Time was set as a repeated effect with an unstructured covariance matrix. Piecewise analysis was used to best account for differing independent variables in the intervention and follow-up periods. The following fixed effects were used in the final models: time (pre- and postprogram in pre-to-post models, postprogram, and 3-month follow-up in follow-up models), intervention condition, the number of exercise therapy sessions attended, and gender. In addition, each fixed effect was used in an interaction term with time, along with a three-way Time × Gender × Intervention condition interaction. All variables were dummy coded except for sessions attended, which were used continuously. Outcomes for longitudinal models included: MADRS, PHQ-9, GAD-7, PAS, NAS, RSES-4, and NPRS.

For within-session analysis, intercept, time (pre- to postsession), week of session, and a crossed effect of Time × Week of session were set as random slopes by participant with a first-order autoregressive covariance matrix. Time × Week of session was set as a repeated effect of subject and used a compound symmetry covariance matrix. Fixed effects in the final models included time (pre- and postsession), intervention condition, week of exercise session, and gender. All fixed effects were also used in interactions with time, as well as a three-way interaction of Time × Gender × Intervention condition. Outcomes for session models included the PHQ-4 and PAS.

The parent study found that concurrent medication use, concurrent outpatient mental health treatment, preprogram activity levels, and the number of yoga sessions attended were all nonsignificant factors [21,22]. Thus, in the current subanalysis of the same data, these variables were not included in the final models. Please see Walter, Otis, Ray, et al., (2023) for more information [22].

## 3. Results

### 3.1. Descriptive Statistics

Of the total sample (*N* = 96), 48% identified as men and 52% as women. Service members were predominantly White (42%), with an average age of 28 years (*SD* = 5.6). Across participants, 71% and 83% were receiving concurrent pharmacotherapy or psychotherapy, respectively, over the course of their participation. In total, 93% of participants were receiving some type of additional mental health treatment during their participation in the NMCSD programs.

The two gender groups were allocated similarly across Surf and Hike Therapies (*p* = 0.220). On average, both women and men attended 3.9 sessions (*p* = 0.981), and dropout rates did not significantly differ between the genders (men = 31%, women = 15%; *p* = 0.079). There were no significant differences by gender on any demographic or preprogram scores, with the exception of the GAD-7, where on average, women endorsed higher anxiety (*MD* = 2.50, *p* = 0.015). Although statistically significant, this difference was not clinically significant [45].

### 3.2. Longitudinal Outcomes within Genders

From pre- to postprogram, participants improved on study outcomes except for pain: MADRS (−6.81, B = *p* < 0.001), PHQ-9 (B = −4.82, *p* < 0.001), GAD-7 (B = −3.54, *p* < 0.001), PAS (B = 2.95, *p* = 0.006), NAS (B = −3.99, *p* <0.001), RSES-4 (B = 1.18, *p* = 0.001), and NPRS (*p* = 0.702). From postprogram to 3-month follow-up, self-reported depression scores on the PHQ-9 (B = −1.39, *p* = 0.023) and resilience scores on the RSES (B = 0.72, *p* = 0.040) improved, while pain scores on the NPRS worsened (B = 0.61, *p* = 0.018). The remaining outcomes did not significantly change during the follow-up period (*p*s = 0.082–0.220).

Statistically significant improvement. When separated by gender, both women and men experienced statistically significant improvements in self-reported anxiety (GAD-7) and clinician-rated and self-reported depression scores (MADRS and PHQ-9) from pre- to postprogram (Table 2). However, among these measures, only clinician-rated depression scores (MADRS) for women changed from postprogram to 3-month follow-up. Otherwise, there were no other statistically significant changes for either gender for self-reported anxiety, self-reported depression, and clinician-rated depression scores during the follow-up period (Table 3).

Across all other domains (i.e., positive affect, negative affect, resilience, and pain) from pre- to postprogram, both women and men experienced statistically significant improvements in negative affect and resilience (NAS and RSES-4). Men also reported significantly increased positive affect (PAS) at postprogram. Neither men nor women reported significant reductions in pain. During the follow-up period, there were no statistically significant improvements in any of these measures, except for continued improvement in resilience among women.

Clinically meaningful improvement. In addition to statistically significant improvement, women experienced clinically significant improvements in self-reported anxiety (GAD-7) and clinician-rated and self-reported depression scores (MADRS and PHQ-9) at postprogram. Although changes in these domains were statistically significant for men, they did not reach the clinically meaningful threshold for anxiety or depression (see Table 2).

### 3.3. Differences in Outcomes by Gender

Women and men attending Surf and Hike Therapies only differed on three outcomes over specific time periods. First, from pre- to postprogram, women experienced a greater decrease in anxiety scores (2.26 points) compared to men (Time × Gender, *p* = 0.029); however, this difference was not clinically significant [45]. As noted with raw severity scores, women began the program with significantly higher scores than men (MD = 2.50).

Second, from pre- to postsession, both women and men experienced statistically significant improvements in depression and positive affect. Women’s depression/anxiety scores decreased 0.93 points more compared to men (Time × Gender, *p* = 0.005). Lastly, women improved 3.73 points more on positive affect following a session compared to men (Time × Gender, *p* = 0.001).

Relatedly, over the course of a session, there was also a significant Time × Gender × Exercise Condition interaction for positive affect (*p* = 0.016). This 3-way interaction was driven by the difference in gender following a Hike Therapy session, where women improved their positive affect scores 5.28 points more than men (*p* = 0.001). On all other outcomes across time points, women and men did not show any significant differences; see Table 2 for pre-to-postprogram outcomes, Table 3 for postprogram-to-3-month follow-up outcomes, and Table 4 for pre-to-postsession outcomes.

## 4. Discussion

The use of exercise interventions for the treatment of MDD, such as surf and hike therapies, has grown significantly over the last several decades. These interventions may have particular utility among active duty populations, where stigma against standard psychological interventions is deeply ingrained [52,53] and physical health is prioritized [54]. As an increasing number of service members and veterans turn to these interventions to improve mental health and wellness, more research is needed to identify those who are most likely to benefit.

Results of the parent study revealed statistically and clinically significant improvements in depression severity, MDD diagnoses, and other psychological outcomes for participants in both Surf and Hike Therapy from preprogram through 3-month follow-up and pre- to postsession [21,22]. In the current study, women experienced statistically and clinically meaningful improvements in depression and anxiety scores at postprogram, and these improvements remained stable in the follow-up period. Women also experienced improvement in negative affect and resilience at postprogram, with improvements remaining steady or increasing further at 3-month follow-up. Men experienced significant improvements over the course of the study as well, but these fell just below the clinically meaningful threshold.

These findings also indicated that some outcomes were moderated by gender while others were not. Specifically, women experienced greater improvements from pre- to postprogram in anxiety than men. It should be noted that despite higher preprogram anxiety severity scores for women compared to men in this study, at the postprogram assessment, women and men endorsed nearly identical anxiety scores (10.25 vs. 10.22, respectively). In the follow-up period, both gender groups maintained their improvements in anxiety, and there were no significant differences in the amount of change between women and men. Although the improvement in anxiety for women may be partially explained by the “regression to the mean” phenomenon, it is noteworthy that preprogram gender differences in anxiety severity were eliminated following Surf and Hike Therapy, moving both genders into the mild/moderate range. Surf and Hike Therapies demonstrate reductions in anxiety from preprogram to 3-month follow-up for both genders but showed an enhanced benefit for women during the intervention period.

Similarly, prior study findings showed that participants reported improvements in depression/anxiety and positive affect over a single surf or hike therapy session [21,22]. Results from the current study expanded upon these findings by showing gender differences in both depression/anxiety symptoms and positive affect from pre- to postsession. In particular, women reported greater decreases in depression/anxiety symptoms and greater increases in positive affect over the course of a session. These results support the only other study on gender differences in the immediate effects of exercise therapy, which found that active duty service women reported significantly larger improvements in depression/anxiety and positive affect within sessions compared to service men [30]. Women may benefit more from a single session of surf or hike therapy for several reasons. They may be less impacted by the stigma of mental health treatment, even when in the context of exercise therapies [55,56]. Service women may also prefer therapies offered outside of traditional military health care settings, as it has been reported that veteran women find these treatment settings particularly uncomfortable [57,58]. A recent review reported that the most prominent barriers to traditional care among veteran women include feeling uncomfortable or unwanted in male-dominated facilities and a perceived lack of sensitivity to gender-related needs [59]. Thus, they may engage more in and benefit more from interventions offered outside of traditional military health care environments.

Interestingly, the finding that service women showed greater psychological benefit from exercise therapies than service men contradicts research in non-active duty populations, which suggests men benefit more [32]. Military women may differ from non-military samples in that, on average, service women may be younger, more inclined or pressured to engage in strenuous physical activity, and may experience different kinds of stigma against traditional treatments. These factors may impact exercise intervention outcomes and explain why outcomes diverged compared to studies of civilians. The results of this study may not generalize to non-service women, but more information is needed on gender differences in exercise intervention outcomes in both military and civilian populations.

Study results also demonstrated that gender differences in positive affect were particularly salient among participants in Hike Therapy, where women experienced significant and large improvements compared to men in that condition. Both Surf and Hike Therapy programs incorporated similar elements, such as outdoor activity, being in the natural environment, and socialization. Hike Therapy may have resulted in greater psychological benefits for women in contrast to Surf Therapy because surfing is generally perceived to be a male-dominated sport with a history of marginalization against women [60,61,62], whereas hiking may be a more gender-neutral sport. Furthermore, the novelty or difficulty of surfing may have impacted a sense of mastery or accomplishment that could limit increases in positive affect compared to hiking. Surfing also tends to be a male-dominated activity [60,61], and this may impact how a woman views and engages in the sport. In comparison, hiking may be a more gender-neutral experience. Also, the Surf Therapy program used one-on-one pairing with an instructor. Although we did not track gender matching between participant and instructor, this may have impacted comfort and engagement in Surf Therapy, especially for those with interpersonal trauma histories. For these reasons, hiking may result in greater anxiety reduction for women compared to men. Future research should examine the mechanisms that underlie these gender differences by exercise type.

Although this study demonstrated gender differences in several outcomes, particularly for the immediate benefits of surf and hike therapies, it is important to emphasize the lack of gender differences in many outcomes, especially regarding long-term benefits. This may speak to the psychological benefits of surf and hike therapies across a variety of different populations [63,64], including those with different psychological conditions [65,66,67]. These findings point to the global and transdiagnostic benefits of surf and hike therapies; these interventions may elicit broad psychological benefits that are not specific to a particular symptom, condition, or group.

This study adds to a growing body of research supporting the psychological benefits of exercise therapies by identifying who experiences the greatest benefit from these interventions. Although many outcomes showed comparable improvement for women and men, several outcomes showed an enhanced benefit for women. In particular, anxiety over the course of the program and immediate improvements in depression/anxiety and positive affect following sessions. Study results helped illuminate gender differences in surf and hike therapy outcomes; however, more research is needed to determine the factors that account for these outcomes. For example, exploring mechanisms that may explain why there are gender differences in some outcomes is important and may help determine whether these interventions should be adapted by gender. Further, expanding our understanding of gender differences in exercise intervention outcomes can provide guidance to clinicians for tailoring evidence-based, comprehensive, and individualized treatment plans for service members. Overall, these study findings highlight the importance of continued research on gender differences in exercise intervention outcomes, especially among marginalized or underserved genders within both active duty and civilian populations.

### 4.1. Limitations

Study findings should be interpreted with consideration for several limitations. First, the demographic questionnaire included a dichotomous response option to assess gender (i.e., “man” or “woman”) rather than separately assessing both sex assigned at birth and gender identity. Second, we did not collect data on the gender of Surf/Hike Therapy Program instructors or the gender makeup of each cohort (namely because each cohort included service members not enrolled in the study). These gender-based factors may have influenced outcomes. Third, we relied solely on quantitative instruments to assess psychological outcomes. Ideally, future studies would extend findings by using a mixed-method approach and including more detailed information on the impact of gender-matched groups and/or group leaders. Finally, the study was a pragmatic trial with few exclusion criteria. As a result, most participants were engaged in concurrent psychotherapy or pharmacotherapy. Although this was statistically controlled in previous analyses [21,22], the unique effects of Surf or Hike Therapy versus a confluence of interventions could not be ascertained in longitudinal analyses. However, most of the observed gender differences were derived from the data collected before and after each exercise session, suggesting that the changes experienced are largely due to the impact of these specific interventions.

### 4.2. Strengths

Despite these limitations, there are many study strengths. The pragmatic trial design increases confidence that our findings are generalizable to a broader active duty population and may encourage the expansion of exercise therapies across the Military Health System. Similarly, there is little research on the outcomes of exercise interventions among service members, particularly female service members. Recent research suggests that service members may not respond as well as civilians to evidence-based mental health treatment [68], emphasizing the importance of the development and examination of low-stigma and effective therapies in this population. Lastly, our study population was diverse, with a large percentage of women, as well as a racial and ethnic demographic breakdown that closely resembles that of the U.S. military [36].

## 5. Conclusions

Our results support a growing body of research supporting the utility and effectiveness of surf and hike therapies among active duty service members [21,22,38] and further suggest that service women experience greater reductions in anxiety compared to service men over the course of the study. Within sessions, women experienced greater depression/anxiety and positive affect, replicating prior research on surf therapy among active duty service members [30]. The effect of gender on positive affect was particularly strong in hike therapy, where women experienced significantly greater improvements in positive affect compared to men. Future research should examine possible mechanisms behind these gender differences (e.g., socialization, engagement, and degree of difficulty) to determine whether gender-based adaptations to exercise interventions are beneficial. Military clinicians may find that augmenting traditional therapies with surf or hike therapy may be beneficial, particularly among women, despite assumptions regarding traditional gender roles. Additionally, motivational strategies with attention to facilitators and barriers to sustained engagement in these interventions may be required to receive maximal benefit. Ultimately, these findings underscore the importance of examining gender differences in therapeutic outcomes among military populations, where women represent an underserved group when it comes to health equity research and advocacy [69]. Furthermore, this examination of gender differences in response to surf and hike therapies aims to contribute to the body of research focusing on reducing disparities in health equity across the U.S. military.

## Figures and Tables

**Table 1:** Preprogram sample characteristics.

Characteristic	Total Sample (*N* = 96)	Women (*n* = 50)	Men (*n* = 46)
	*n* (%)	*n* (%)	*n* (%)
Race/ethnicity ^a^			
Asian or Asian-American and Native American or Alaska Native	4 (4.2)	-	-
Black or African American	15 (15.6)	-	-
Hispanic, Latino, or Spanish origin	18 (18.8)	-	-
Multiracial	19 (19.8)	-	-
White	40 (41.7)	-	-
Exercise condition			
Surf	48 (50.0)	22 (45.8)	26 (58.3)
Hike	48 (50.0)	28 (54.2)	20 (41.7)
Rank ^a^			
E1–E4	34 (35.4)	-	-
E5–E9	57 (59.4)	-	-
Officer	5 (5.2)	-	-
Concurrent mental health treatment	89 (92.7)	46 (92.0)	43 (93.5)
Pharmacotherapy	68 (70.8)	35 (70.0)	33 (71.7)
Psychotherapy	80 (83.3)	42 (84.0)	38 (82.6)
Completion of assigned program ^b,c^	68 (77.3)	39 (84.8)	29 (69.0)
	*M (SD)*	*M (SD)*	*M (SD)*
Age, years	28.1 (5.6)	27.5 (4.9)	28.8 (6.3)
Sessions attended ^c,d^	3.9 (1.6)	3.9 (1.6)	3.9 (1.7)
Preprogram measures			
MADRS	27.0 (8.4)	28.0 (9.1)	25.9 (7.6)
PHQ-9	17.1 (4.9)	17.5 (4.7)	16.6 (5.1)
GAD-7	13.8 (5.1)	15.0 (4.2) *	12.5 (5.6) *
PAS	20.6 (7.0)	20.0 (7.3)	21.2 (6.8)
NAS	23.9 (8.0)	25.0 (7.7)	22.6 98.2)
RSES-4	9.6 (3.5)	9.6 (3.5)	9.7 (3.6)
NPRS	3.1 (2.5)	2.9 (2.6)	3.4 (2.3)

Note. E = enlisted rank; GAD-7 = 7-item Generalized Anxiety Disorder scale; MADRS = Montgomery–Åsberg Depression Rating Scale; NAS = Negative Affect Schedule; NPRS = Numerical Pain Rating Scale; PAS = Positive Affect Schedule; PHQ-9 = 9-item Patient Health Questionnaire; RSES-4 = 4-item Response to Stressful Events Scale. Totals may not sum to sample numbers or percentages due to missing data. Asterisks indicate a significant difference between self-identified genders. ^a^ All attempts were made to report race/ethnicity and rank data properly, but due to low cell counts, variables were combined to protect participant identity, and they were not stratified by condition. ^b^ Program completion was defined by the Naval Medical Center San Diego as missing no more than two sessions of the assigned intervention. ^c^ The sudden onset of COVID-19 abruptly ended programming; participants (*n* = 8) in the affected cohort were not counted for completion and attendance variables. ^d^ Only sessions in which the assigned intervention was conducted were included. Occasionally, due to adverse weather, sessions consisted of alternative activities (e.g., visits to the National Surf Museum, hiking strength and conditioning class). * *p* < 0.05.

**Table 2:** Estimated Marginal Means and Group Differences of Time × Gender from pre- to postprogram.

Outcome	EMM		Within-Group Change		Between-Group Difference	
			*MD* (95% CI)	*p*	*MD* (95% CI)	*p*
Pre- to Postprogram						
MADRS						
Women	Pre	27.98	−8.11 (−11.24, −4.98)	<0.001	−2.59 (−7.03, 1.85)	0.256
	Post	19.88				
Men	Pre	26.17	−5.52 (−8.77, −2.27)	0.001		
	Post	20.66				
PHQ-9						
Women	Pre	17.46	−5.05 (−6.91, −3.20)	<0.001	−0.46 (−3.10, 2.17)	0.733
	Post	12.41				
Men	Pre	16.84	−4.59 (−6.52, −2.67)	<0.001		
	Post	12.25				
GAD-7						
Women	Pre	14.92	−4.68 (−6.09, −3.26)	<0.001	−2.26 (−4.26, −0.26)	0.029
	Post	10.25				
Men	Pre	12.64	−2.41 (−3.87, −0.96)	0.001		
	Post	10.22				
PAS						
Women	Pre	20.11	2.26 (−0.62, 5.13)	0.122	−1.39 (−5.49, 2.71)	0.509
	Post	22.37				
Men	Pre	20.79	3.64 (0.64, 6.65)	0.018		
	Post	24.43				
NAS						
Women	Pre	24.77	−4.94 (−7.05, −2.84)	<0.001	−1.91 (−4.92, 1.11)	0.218
	Post	19.82				
Men	Pre	23.07	−3.04 (−5.25, −0.82)	0.008		
	Post	20.03				
RSES-4						
Women	Pre	9.61	1.34 (0.37, 2.32)	0.007	0.32 (−1.07, 1.71)	0.651
	Post	10.96				
Men	Pre	9.59	1.02 (0.01, 2.04)	0.048		
	Post	10.61				
NPRS						
Women	Pre	2.89	−0.16 (−0.71, 0.39)	0.567	−0.17 (−0.95, 0.62)	0.681
	Post	2.73				
Men	Pre	3.37	0.01 (−0.57, 0.58)	0.984		
	Post	3.38				

Note. EMM = estimated marginal mean; MD = mean difference; MADRS = Montgomery–Åsberg Depression Rating Scale; PHQ-9 = 9-item Patient Health Questionnaire; GAD-7 = 7-item Generalized Anxiety Disorder Scale; PAS = Positive Affect Schedule; NAS = Negative Affect Schedule; RSES-4 = 4-item Response to Stressful Events Scale; NPRS = Numerical Pain Rating Scale. EMMs were derived from multilevel models that included: time (pre- to postprogram), exercise condition, number of sessions attended, and gender.

**Table 3:** Estimated Marginal Means and Group Differences of Time × Gender from postprogram to 3-month follow-up.

Outcome	EMM		Within-Group Change		Between-Group Difference	
			*MD* (95% CI)	*p*	*MD* (95% CI)	*p*
Postprogram to 3-Month Follow-Up						
MADRS						
Women	Post	19.87	−3.34 (−6.34, −0.34)	0.029	−3.37 (−7.55, 0.82)	0.119
	3mo	16.53				
Men	Post	20.53	0.03 (−2.99, 3.05)	0.986		
	3mo	20.56				
PHQ-9						
Women	Post	12.37	−1.36 (−3.02, 0.30)	0.107	0.06 (−2.28, 2.40)	0.959
	3mo	11.01				
Men	Post	12.16	−1.42 (−3.13, 0.29)	0.102		
	3mo	10.74				
GAD-7						
Women	Post	10.14	0.06 (−1.44, 1.55)	0.941	1.44 (−0.66, 3.54)	0.184
	3mo	10.20				
Men	Post	10.20	−1.38 (−2.91, 0.14)	0.075		
	3mo	8.82				
PAS						
Women	Post	22.18	2.20 (−0.35, 4.74)	0.090	1.69 (−1.90, 5.28)	0.359
	3mo	24.37				
Men	Post	24.77	0.51 (−2.11, 3.12)	0.702		
	3mo	25.28				
NAS						
Women	Post	20.02	−1.86 (−3.98, 0.27)	0.086	−1.01 (−4.01, 1.98)	0.510
	3mo	18.17				
Men	Post	19.89	−0.84 (−3.03, 1.35)	0.445		
	3mo	19.05				
RSES-4						
Women	Post	10.92	1.00 (0.06, 1.96)	0.038	0.58 (−0.76, 1.93)	0.399
	3mo	11.92				
Men	Post	10.53	0.43 (−0.56, 1.41)	0.390		
	3mo	10.96				
NPRS						
Women	Post	2.73	0.54 (−0.16, 1.24)	0.130	0.18 (−4.80, 5.16)	0.780
	3mo	3.27				
Men	Post	3.28	0.68 (−0.04, 1.40)	0.065		
	3mo	3.96				

Note. EMM = estimated marginal mean; MD = mean difference; MADRS = Montgomery–Åsberg Depression Rating Scale; PHQ-9 = 9-item Patient Health Questionnaire; GAD-7 = 7-item Generalized Anxiety Disorder Scale; PAS = Positive Affect Schedule; NAS = Negative Affect Schedule; RSES-4 = 4-item Response to Stressful Events Scale; NPRS = Numerical Pain Rating Scale. EMMs were derived from multilevel models that included: time (postprogram to 3-month follow-up), exercise condition, number of sessions attended during the follow-up period (if applicable), and gender.

**Table 4:** Estimated Marginal Means and Group Differences of Time × Gender from pre- to postsession.

Outcome	EMM		Within-Group Change		Between-Group Difference	
			*MD* (95% CI)	*p*	*MD* (95% CI)	*p*
Pre- to Postsession						
PHQ-4						
Women	Pre	6.14	−3.45 (−3.89, −3.01)	<0.001	−0.93 (−1.57, −0.29)	0.005
	Post	2.69				
Men	Pre	5.69	−2.52 (−2.99, −2.05)	<0.001		
	Post	3.17				
PAS						
Women	Pre	23.35	10.28 (8.77, 11.80)	<0.001	3.73 (0.98, 6.48)	0.001
	Post	33.63				
Men	Pre	24.43	6.55 (4.95, 8.16)	<0.001		
	Post	30.99				

Note. EMM = estimated marginal mean; MD = mean difference; PHQ-4 = 4-item Patient Health Questionnaire; PAS = Positive Affect Schedule. EMMs were derived from multilevel models that included: time (pre- to postsession), exercise condition, week of session, and gender.

## Data Availability

The datasets generated and/or analyzed during the current study are not publicly available due to security protocols and privacy regulations, but they may be made available on reasonable request by the Naval Medical Center San Diego or Naval Health Research Center Institutional Review Board (contact phone: +1-619-553-8400).
